# Pioglitazone Enhances the Beneficial Effects of Glucocorticoids in Experimental Nephrotic Syndrome

**DOI:** 10.1038/srep24392

**Published:** 2016-05-04

**Authors:** S. Agrawal, M. A. Chanley, D. Westbrook, X. Nie, T. Kitao, A. J. Guess, R. Benndorf, G. Hidalgo, W. E. Smoyer

**Affiliations:** 1 Center for Clinical and Translational Research, The Research Institute at Nationwide Children’s Hospital, Columbus, OH, USA; 2 James and Connie Maynard Children’s Hospital, Greenville, NC, USA; 3 Department of Pediatrics, College of Medicine, The Ohio State University, Columbus, OH, USA; 4 Department of Pediatrics, Brody School of Medicine, East Carolina University, Greenville, NC, USA.

## Abstract

Glucocorticoids are the primary therapy for nephrotic syndrome (NS), but have serious side effects and are ineffective in ~20–50% of patients. Thiazolidinediones have recently been suggested to be renoprotective, and to modulate podocyte glucocorticoid-mediated nuclear receptor signaling. We hypothesized that thiazolidinediones could enhance glucocorticoid efficacy in NS. We found that puromycin aminonucleoside-induced proteinuria in rats was significantly reduced by both high-dose glucocorticoids (79%) and pioglitazone (61%), but not low-dose glucocorticoids (25%). Remarkably, pioglitazone + low-dose glucocorticoids also reduced proteinuria (63%) comparably to high-dose glucocorticoids, whereas pioglitazone + high-dose glucocorticoids reduced proteinuria to almost control levels (97%). Molecular analysis revealed that both glucocorticoids and pioglitazone enhanced glomerular synaptopodin and nephrin expression, and reduced COX-2 expression, after injury. Furthermore, the glomerular phosphorylation of glucocorticoid receptor and Akt, but not PPARγ, correlated with treatment-induced reductions in proteinuria. Notably, clinical translation of these findings to a child with refractory NS by the addition of pioglitazone to the treatment correlated with marked reductions in both proteinuria (80%) and overall immunosuppression (64%). These findings together suggest that repurposing pioglitazone could potentially enhance the proteinuria-reducing effects of glucocorticoids during NS treatment.

Nephrotic syndrome (NS) is among the most common kidney diseases affecting both children and adults. Oral glucocorticoids (GCs) have been the primary therapy for NS in both children and adults for >60 years, although GCs have serious side effects and are clinically ineffective in ~20% of children and ~50% of adults^1^,^2^. Unfortunately most alternative agents are only partially effective, and are associated with significant side effects^3^. Thus, there is a critical need for both more effective and less toxic treatments for NS.

In this context, the FDA-approved type II diabetes drugs, thiazolidinediones (TZDs), have been shown in large observational studies to slow the progression of diabetic nephropathy and to reduce proteinuria, potentially due to their direct renoprotective effects in addition to their known promoting effects on insulin sensitivity^4^. Although the mechanism of action for such protection remains unclear, recent laboratory studies have reported partial protective effects of TZDs in animal models of nephropathy^5^,^6^,^7^,^8^, as well as in direct podocyte injury *in vitro*^9^,^10^,^11^,^12^. Notably, both GCs and TZDs are known to act via a similar molecular mechanism by binding to their respective nuclear receptors [glucocorticoid receptor (GR) and peroxisome proliferator-activated receptor gamma (PPARγ), respectively], both of which belong to the nuclear hormone receptor superfamily^13^,^14^. We recently reported direct protective effects of GCs and TZDs on podocytes, potentially due to crosstalk between the GC and TZD nuclear receptor-mediated signaling pathways, as demonstrated by the ability of TZDs to modulate the GC signaling pathway^10^. We thus hypothesized that nuclear receptor crosstalk between podocyte TZD and GC pathways could be exploited to enhance the proteinuria-reducing effects of GCs during NS treatment. To test this hypothesis we: 1) Determined the ability of pioglitazone to protect and enhance the efficacy of GCs in reducing proteinuria in puromycin aminonucleoside (PAN)-induced NS, and 2) Analyzed the potential mechanistic pathways for protection in the glomeruli of rats with PAN-induced NS treated with combinations of GC and/or the TZD pioglitazone. and 3) Analyzed the potential of pioglitazone to enhance GC efficacy in reducing proteinuria in a child with refractory NS.

## Results

### Glucocorticoids and Pioglitazone Both Reduce PAN-Induced Proteinuria in Rats

**Figure 1:**
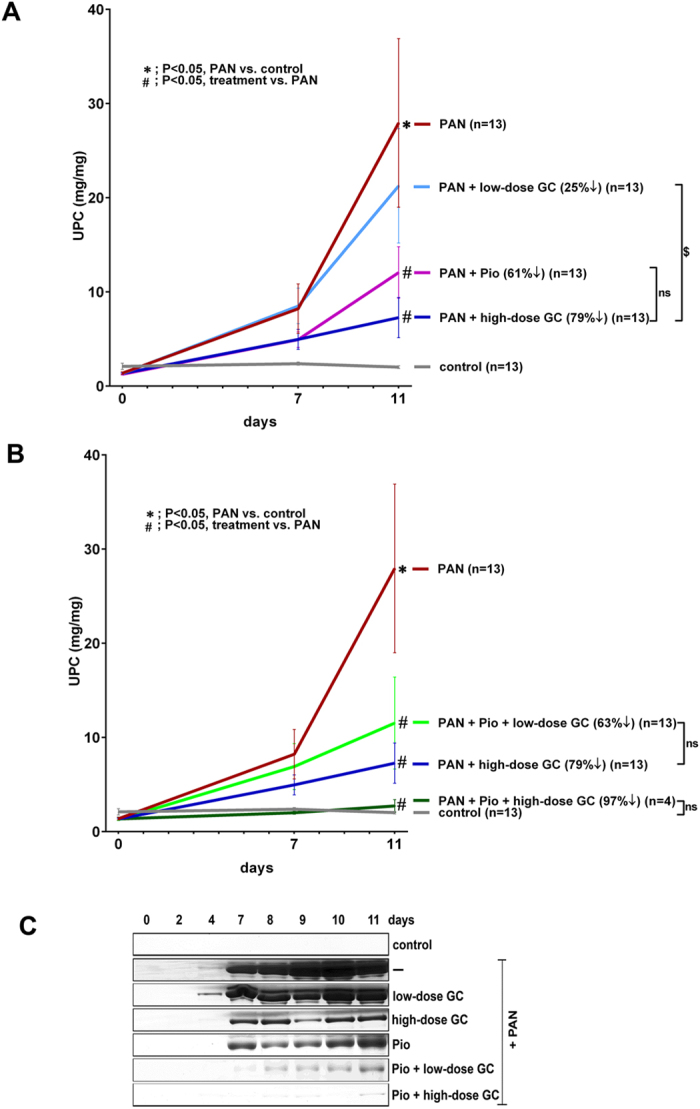
Pioglitazone Reduces PAN-Induced Proteinuria and Enhances the Protective Effects of Glucocorticoids. Proteinuria was induced in male Wistar rats by single intravenous PAN injections (50 mg/kg) on Day 0. Treatment groups also received: (**A**) low-dose GC (5 mg/kg; n = 13), high-dose GC (15 mg/kg; n = 13), and pioglitazone (Pio; 10 mg/kg; n = 13); and (**B**) high-dose GC (15 mg/kg; n = 13) and combinations of pioglitazone with low-dose GC (Pio + low-dose GC; n = 13) or high-dose GC (Pio + high-dose GC; n = 4) for 10 days. Urinary protein/creatinine ratios (UPC) are plotted through the course of the experiment (Mean ± SEM; *P < 0.05, PAN vs. control; ^#^P < 0.05, treatment vs. PAN; ^ns^P > 0.05; ^$^P < 0.05; determined by Two-way ANOVA tukey’s multiple comparison test). (**C**) Representative gels depicting proteinuria induction after PAN injection and reduction after treatments with GC and pioglitazone. Massive amounts of urinary albumin after PAN injection were reduced by treatment with high-dose GC and pioglitazone, and even more notably reduced by treatment with pioglitazone combined with low-dose GC and high-dose GC (Pio + low-dose GC and Pio + high-dose GC). Urines were collected daily and equal volumes (4 μl) from selected days were analyzed by SDS–polyacrylamide gel electrophoresis and Coomassie Brilliant Blue staining.

Male Wistar rats developed massive proteinuria after a single IV PAN injection of 50 mg/kg, which peaked on day 11 (UPC 28.0 ± 9.0 mg/mg) (Fig. 1A,C). The control rats received IV saline and maintained baseline levels of urinary protein (UPC 2.0 ± 0.1 mg/mg). To analyze the ability of GCs to reduce PAN-induced proteinuria, 13 rats/group were also treated with intraperitoneal (IP) daily administration of low-dose GC (5 mg/kg), which did not result in a significant reduction in proteinuria (25%, *P* = NS vs. PAN; UPC 21.3 ± 6.0 mg/mg). In contrast, high-dose GC (15 mg/kg) reduced proteinuria significantly (79%, *P < *0.05 vs. PAN; UPC 7.3 ± 2.1 mg/mg) (Fig. 1A,C). Furthermore, enteral daily administration of pioglitazone (10 mg/kg) also resulted in comparable proteinuria reduction (61%, *P* = <0.05 vs. PAN; UPC 12.1 ± 2.7 mg/mg) (Fig. 1A,C). The proteinuria values are also indicated in tabular form at three different time points in Table 1. In summary, GC treatment resulted in a reduction in PAN-induced proteinuria, but only at a high dose, while pioglitazone treatment alone also resulted in proteinuria reduction.

Protective effects of GCs and pioglitazone were also demonstrated in cultured podocytes by restoring the actin cytoskeletal arrangement and cell viability (see Supplementary Figure S1).

### Pioglitazone Enhances the Efficacy of Glucocorticoids in Reducing PAN-Induced Proteinuria

Administration of pioglitazone combined with low-dose GC to PAN-injected rats resulted in further reduction in proteinuria at day 11 (63%, *P < 0.05* vs. PAN; UPC 11.5 ± 4.9 mg/mg) than with pioglitazone alone (Fig. 1B,C). This proteinuria reduction was also statistically similar to the proteinuria reduction induced by high-dose GC (79%; *P* = NS, pioglitazone + low-dose GC vs. high-dose GC), despite a 67% reduction in GC dosing (15 mg/kg vs. 5 mg/kg). In addition, treatment with pioglitazone combined with high-dose GC reduced proteinuria even more, achieving proteinuria reductions that were statistically similar to control levels (97%, *P* = NS vs. control; UPC 2.7 ± 0.7 mg/mg) (Fig. 1B,C). The proteinuria values are also indicated in tabular form at three different time points in Table 1. Moreover, the addition of pioglitazone to either low-dose or high-dose GC did not induce changes in body weight or kidney weight (that might be suggestive of edema, a known side effect) compared to treatment with GC alone, (see Supplementary Table S1). In summary, pioglitazone enhanced the efficacy of low-dose GC in reducing proteinuria, enabling comparable reductions in proteinuria to that of high-dose GC despite a 67% reduction in GC dosing. Moreover, the addition of pioglitazone to high-dose GC was able to further enhance the efficacy of high-dose GC (97% vs. 79% with high dose-GCs alone), thereby enabling enhanced proteinuria reduction to essentially normal control levels.

### Pioglitazone and Glucocorticoids Both Restore Synaptopodin Expression in PAN-Injured Glomeruli

**Figure 2:**
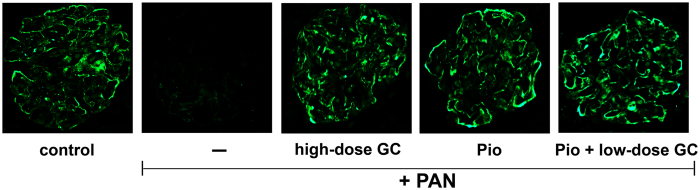
Pioglitazone and Glucocorticoids Restore Synaptopodin Expression in PAN-Injured Glomeruli. The panels show representative images of the immunofluorescence staining for synaptopodin in the glomeruli of kidneys from control, PAN-injured and PAN-injured rats treated with high-dose GC, Pio, and Pio + low-dose GC.

Evaluation of glomeruli by immunofluorescence microscopy revealed that PAN-induced injury resulted in marked reductions in glomerular synaptopodin protein expression, which was restored after treatment with high-dose GC, pioglitazone, and pioglitazone + low-dose GC (Fig. 2). These results thus revealed that glomerular synaptopodin expression correlated inversely with proteinuria (see Figs 1 and 2 and Table 1).

### Pioglitazone and Glucocorticoids Both Enhance the Glomerular Expression of Podocyte Markers and Reduce COX-2 Expression

**Figure 3:**
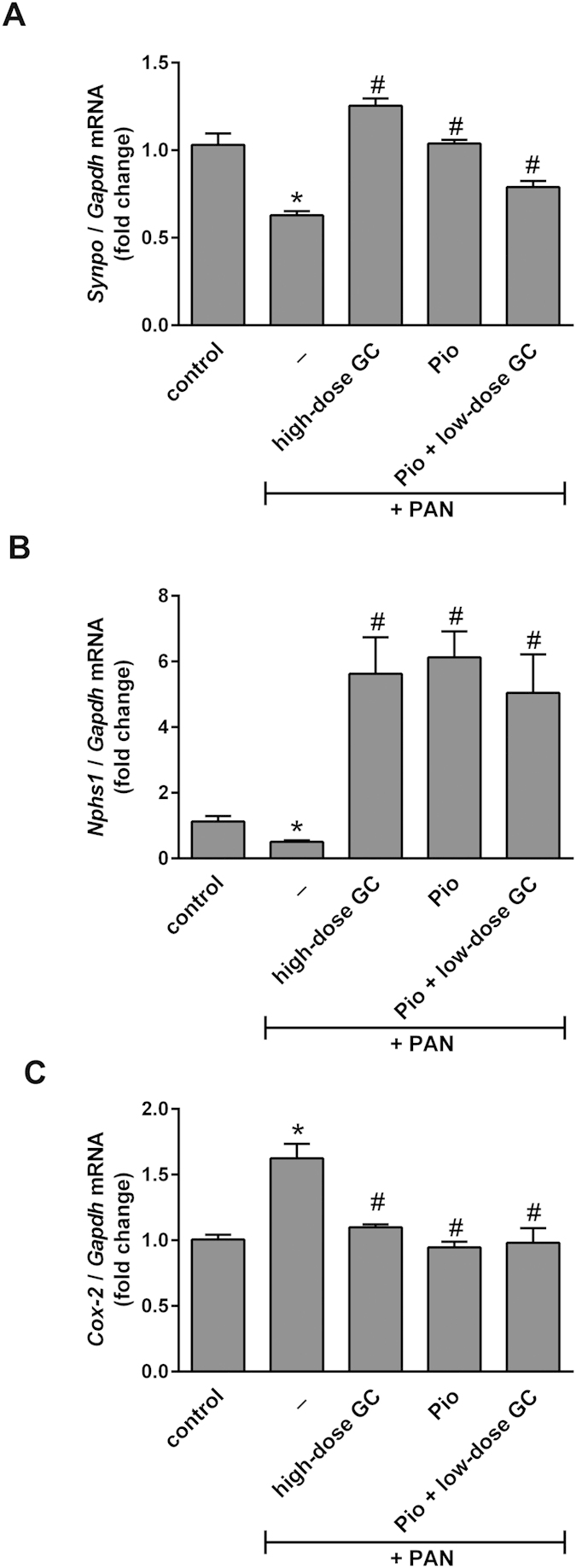
Pioglitazone and Glucocorticoids Enhance the Glomerular Expression of Podocyte Markers and Reduce COX-2 Expression. Total RNA was extracted from glomeruli isolated from control, PAN-injected, and PAN-injected rats treated with high-dose GC, Pio, and Pio + low-dose GC (n = 3 per group). Relative mRNA levels of (**A**) *Synpo*, (**B**) *Nphs1* and (**C**) *Ptgs2* were measured by quantitative reverse transcription–polymerase chain reaction and normalized to *Gapdh.* Data are presented as Mean ± SEM (*P < 0.05 vs. control; ^#^*P* < 0.05 vs. PAN; determined by *t*-test).

Glomerular mRNA expression of *Synpo* (encoding for synaptopodin) and *Nphs1* (encoding for nephrin) were both reduced after PAN-induced injury, but were restored after treatment with high-dose GC, pioglitazone and pioglitazone + low-dose GC (Fig. 3A,B). In contrast, glomerular mRNA expression of *Ptgs2* (encoding for COX-2) was increased by PAN injury but also restored to normal levels after treatment with high-dose GC, pioglitazone, and pioglitazone + low-dose GC (Fig. 3C).

### Phosphorylation of Glomerular Glucocorticoid Receptor and Akt, but not PPARγ, Correlate with Glucocorticoid- and Pioglitazone-Induced Reductions in Proteinuria

**Figure 4:**
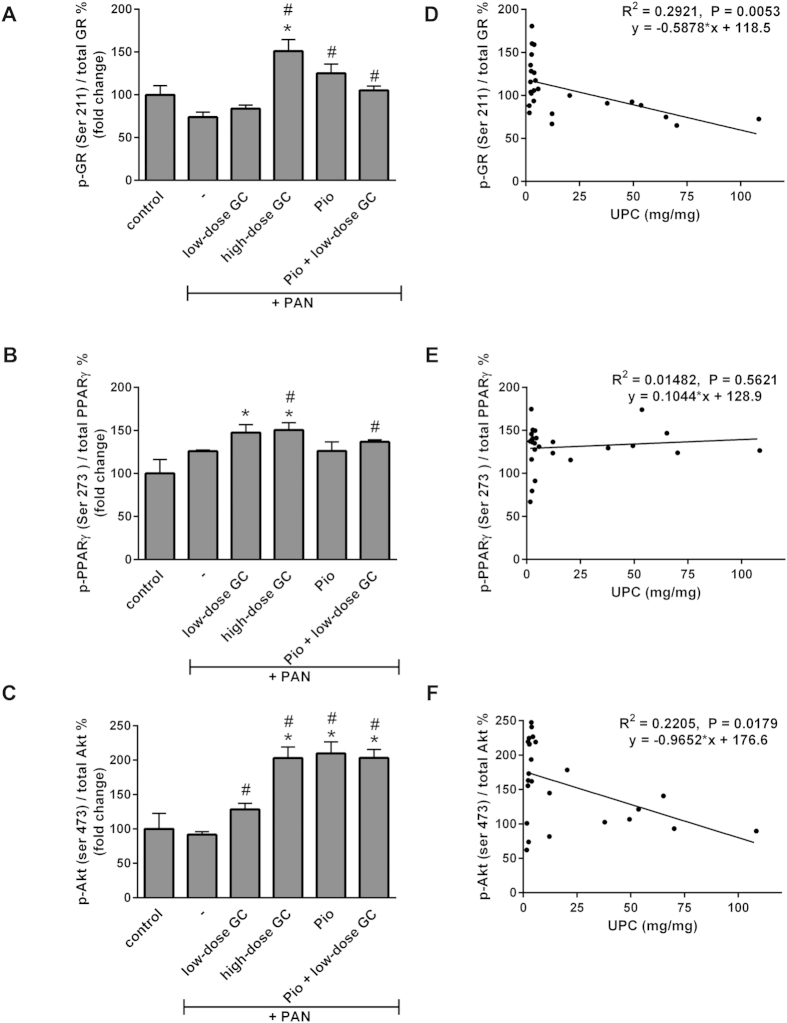
Phosphorylation of the Glomerular Glucocorticoid Receptor and Akt, but not PPARγ, Correlate with Glucocorticoid- and Pioglitazone-Induced Reductions in Proteinuria. Total protein was extracted from glomeruli isolated from control, PAN-injected, and PAN-injected rats treated with low-dose GC, high-dose GC, Pio and Pio + low-dose GC (n = 4 per group). Relative percentage phosphorylation of glucocorticoid receptor (GR), PPARγ and Akt was measured by western blotting and densitometry analyses. Values were plotted as Mean ± SEM (*P < 0.05 vs. control; ^#^*P* < 0.05 vs. PAN; determined by *t*-test) distributed by treatment groups (**A–C**). Percentage phosphorylation was also plotted against the individual proteinuria values and correlations were computed by Pearson correlation coefficients (**D–F**).

Glomerular activation of the GR, as indicated by phosphorylation (p-GR) at Ser 211, was induced by treatment with high-dose GC, pioglitazone and pioglitazone + low-dose GC, but not with low-dose GC alone (Fig. 4A). Moreover, the levels of glomerular GR activation/phosphorylation correlated inversely with proteinuria in PAN-injected and treated rats (*P* = 0.005; Fig. 4D). On the contrary, neither GCs nor pioglitazone blocked the phosphorylation of PPARγ at Ser 273, it’s known phosphorylation site (Fig. 4B). Moreover, the level of PPARγ phosphorylation (p-PPARγ at Ser 273) did not show any correlation with proteinuria (Fig. 4E). Furthermore, evaluation of Akt activity as measured by phosphorylation (p-Akt) at Ser 473 was increased in all the treatment groups, and the level of phosphorylation correlated inversely with proteinuria in PAN-injected and treated rats (*P* = 0.018; Fig. 4C,F). In summary, p-GR and p-Akt, but not p-PPARγ levels inversely correlate with proteinuria in PAN-injected and treated rats.

## Discussion

These studies were designed to test the hypothesis that crosstalk between podocyte TZD and GC signaling pathways can be exploited to enhance the proteinuria-reducing effects of GCs during NS treatment. Our results revealed that pioglitazone alone reduced proteinuria during experimental NS in rats, and also enhanced the efficacy of GC in reducing proteinuria. Furthermore, combining pioglitazone treatment with low-dose GC enabled a 67% reduction in GC dosing without any loss of efficacy in reducing proteinuria. In addition, combining pioglitazone with high-dose GC notably enhanced the proteinuria-reducing effects of GC to almost normal control levels. From a mechanistic perspective, these protective effects were associated with enhancements in podocyte markers (Synaptopodin and Nephrin) and a reduction in COX-2 expression in glomeruli. Moreover, glomerular phosphorylation of the GR and Akt, but not PPARγ, correlated inversely with proteinuria in both PAN-injured and treatment groups. Finally, translation of these findings to a child with refractory NS revealed that the addition of pioglitazone to the treatment regimen was associated with marked reductions in both proteinuria (80%) and overall immunosuppression (64%) (see Supplementary Figures S2 and S3). These findings together suggest that repurposing pioglitazone could potentially enhance the proteinuria-reducing effects of GCs during NS treatment.

Although GCs have been used as the primary therapy for NS for the past 60 years, it was not until 20–30 years after their introduction that GCs were first reported to reduce proteinuria in animal disease models^15^. A few recent reports have also noted renoprotective effects for pioglitazone alone in NS models^6^,^7^. However, our study attempted to significantly extend these findings to compare the *in vivo* proteinuria-reducing effects of pioglitazone alone to that of GC alone, as well as to pioglitazone + low- and high-dose GC combinations. Notably, these findings suggest that the addition of pioglitazone may enable a 67% reduction in GC dosing (and thus reduced toxicity) without any loss in clinical efficacy in proteinuria reduction. Moreover, the addition of pioglitazone might also potentially be able to enhance the clinical efficacy of higher doses of GCs, and could thus also have utility in patients with steroid resistant NS. Importantly, in contrast to other TZD members like rosiglitazone in the anti-diabetic class of drugs, meta-analysis studies have revealed no increased cardiovascular risk due to pioglitazone, and it continues to be widely used in the treatment of diabetes^13^.

Synaptopodin is an actin-associated protein in podocyte foot processes and its expression is known to inversely correlate with glomerular injury during NS^16^,^17^. In addition, the expression of another podocyte marker Nephrin (*Nphs1*) is also known to be altered during the development of proteinuria^18^. In the present study, we observed restored and increased glomerular expression of both synaptopodin and nephrin upon treatment with pioglitazone and GC after injury. Notably, nephrin expression was increased robustly upon treatment with both GC and pioglitazone, which is possibly due to the direct effects of these drugs on the transcription of nephrin mRNA via the GC response elements (GRE) and peroxisome proliferator response elements (PPRE), respectively, in the promoter region of nephrin gene^19^,^20^. We have also extended our previously-reported correlation between podocyte *Ptgs2* (COX-2) expression and *in vitro* podocyte injury and recovery seen after treatment with pioglitazone and GC to an *in vivo* model of NS in glomeruli of rats injured with PAN and treated with pioglitazone and GC^21^. Since COX-2 has been widely reported to be involved in glomerular injury, and is known to be regulated by trans-repression activities of both GR and PPARγ^22^, our results provide additional mechanistic insight into the protective pathways induced in podocytes by pioglitazone and GC treatment during NS.

Upon ligand binding, GR undergoes rapid phosphorylation at Ser 211 which is associated with its activation^23^. We have previously reported the ability of pioglitazone, like GCs (although to a lower degree), to modulate GR phosphorylation in podocytes^10^. Extension of these findings in the current study to glomeruli in an *in vivo* model of NS treated with GC and pioglitazone revealed that the proteinuria reductions are directly correlated with GR phosphorylation at this site. Furthermore, since PPARγ is the canonical receptor of pioglitazone, we also studied the status of its phosphorylation at Ser 273 upon treatment with GC and pioglitazone. Phosphorylation at this site is believed to be mediated by Cdk5, which has been shown to be blocked by PPARγ agonists, leading to their obesity-related effects^24^. In the current study, we did not find any correlation between phosphorylation levels of PPARγ at Ser 273 and proteinuria. This however, does not rule out the role of PPARγ in mediating protective effects during NS or renal injury, as has been reported by others^8^,^25^. Finally, the crucial role of Akt2 activation by mammalian target of rapamycin complex 2 (mTORC2) in podocyte survival has been recently emphasized in a mouse model of podocyte-specific deletion of Akt, as well as in human chronic kidney disease^26^. Therefore, we analyzed the relevance of this pathway in PAN-induced NS and treatment by pioglitazone and GC by measuring the activation of Akt, as indicated by its phosphorylation at Ser 473 by mTORC2. We not only found that the treatment groups had increased glomerular p-Akt compared to the PAN-injured group, but also that glomerular p-Akt levels correlated inversely with proteinuria, thus further underscoring the apparent role of this pathway in glomerular disease.

In addition to NS, GCs are the first line of therapy for many other diseases, including rheumatoid arthritis, asthma and many inflammatory conditions^27^,^28^. Similarly, there is an emerging role for pioglitazone and other TZDs as novel therapeutic agents for cancer, inflammatory diseases, and arthritis^29^,^30^. Our data now identify clear beneficial effects from combining pioglitazone with GCs in treating glomerular disease, which may be due to crosstalk between TZD- and GC-mediated pathways in podocytes. Others have also recently reported protective effects of combinations of pioglitazone and GCs in other disease models such as inflammation, arthritis and lung cancer^27^,^28^,^31^. Indeed, combination drug therapy is a common practice in medicine, and often results in improved therapeutic effects compared to monotherapy.

Finally, the translation of the results detailed above for the first time to a child with refractory NS is provocative, albeit anecdotal (see Supplementary Figures S2 and S3). While a single case report certainly cannot imply cause and effect, the addition of pioglitazone to the treatment regimen of the child with refractory NS was associated with clinically important improvements in his condition. After experiencing eight hospitalizations and twenty five IV albumin infusions, and treatment with five relatively toxic immunosuppressive medications over two years, initiation of pioglitazone was associated with a subsequent 80% decrease in proteinuria over the ensuing months (without a decrease in eGFR), completely eliminating the need for any subsequent hospitalizations or albumin infusions. Moreover, this improvement enabled his treatment team to reduce his overall immunosuppression by 64%, thereby markedly reducing his ongoing exposure to potentially toxic medications and reducing his risk for infection (see Supplementary Tables S2A and S2B). While he did not enter into complete remission, the combination of elimination of hospitalizations with the marked reduction in overall immunosuppression has greatly enhanced his quality of life, reduced his healthcare utilization and expenses, and potentially reduced his risk for progression to end-stage kidney disease. Furthermore, the model used in this study for the calculation of overall immunosuppression (Total ISS) could easily be personalized for other patients or diseases based on the specific immunosuppressive medications prescribed.

These studies had a few notable limitations. First, the experiments were designed to determine if pioglitazone is able to enhance the ability of GC to protect against proteinuria, and not specifically to analyze its ability to enhance resolution of existing proteinuria. In addition, the studies were only designed to analyze short term effects of pioglitazone and GC on proteinuria reduction, and not their ability to induce prolonged reductions in proteinuria.

In summary, the protective effects of pioglitazone and GC reported here in an *in vivo* model of NS, combined with the clinical improvements seen in the above child, suggest two intriguing possibilities: 1) That pioglitazone could potentially be used to enable reduced dosing (and toxicity) of GC without a reduction in clinical efficacy, and 2) That pioglitazone could potentially be used to enhance the clinical efficacy of GCs in the setting of clinical steroid resistance. Hopefully, a future prospective clinical trial will enable validation of the potential repurposing of pioglitazone as a GC supplement for NS, and possibly other diseases treated with GCs.

## Methods

### Animal Study Design

The performed studies were approved by the Institutional Animal Care and Use Committee at Nationwide Children’s Hospital and all the experiments were performed in accordance with the approved guidelines. Proteinuria was induced in male Wistar rats weighing ~150–200 g by single intravenous (IV) PAN (Sigma-Aldrich, St. Louis, MO) injection (50 mg/kg) on Day 0, while the control group received IV saline injections (n = 13). A group of PAN injected rats (n = 13) were left untreated and received sham oral gavage vehicle solution (0.5% methylcellulose, 0.025% Tween-20), while the treatment groups (n = 13/group) also received: (i) Low-dose GC (Methylprednisolone; 5 mg/kg; n = 13; Solu-Medrol, Pfizer Inc., New York, NY) by intraperitoneal (IP) injection daily, (ii) High-dose GC (15 mg/kg; n = 13), (iii) Pioglitazone (Pio; 10 mg/kg; n = 13; Actos; Takeda, Deerfield, IL) by oral gavage daily, (iv) Combined low-dose GC + pioglitazone [(Pio + low-dose GC; n = 13), and (v) Combined high-dose GC + pioglitazone (Pio + high-dose GC; n = 4)] starting at Day 0. Two control rats also received pioglitazone alone (10 mg/kg) by oral gavage daily with no PAN injection. Urine samples were collected at baseline (prior to injury) and daily during the study for analysis. The rats were weighed daily and sacrificed on Day 11 at the point of expected peak proteinuria and the kidneys harvested, weighed and processed for paraffin sectioning and glomerular isolation.

### Urine Analyses

Albuminuria was monitored by analyzing 4 μl urine samples taken at Days 0, 2, 4, 7, 8, 9, 10 and 11 for the presence of albumin by SDS-PAGE and Coomassie Brilliant Blue staining. Urine protein:creatinine ratios (UPC) were measured by Antech Diagnostics GLP (Morrisville, NC), which is fully compliant with Good Laboratory Practice (GLP) regulations.

### Glomerular Isolation

Glomeruli were isolated using a standard sequential sieving method, as described previously^21^,^32^ and the resulting preparations were washed with PBS and assessed under a light microscope, which confirmed ~95% glomeruli. One half of the glomerular preparation was suspended in RLT buffer (Qiagen, Germantown, MD) containing β-Mercaptoethanol (Sigma-Aldrich) and homogenized with a 21 gauge needle for RNA isolation as described below. Protein extracts were prepared from the other half of the glomerular preparation in Tissue Protein Extraction Reagent (TPER; Thermo Fisher Scientific, Waltham, MA).

### RNA Extraction and Real Time Polymerase Chain Reaction

Total RNA from isolated glomeruli was isolated using the RNeasy kit (Qiagen) according to the manufacturer’s instructions. The purity and yield of RNA was determined by measuring the absorbance at 260 and 280 nm and used for quantitative reverse transcribed-polymerase chain reaction (qRT-PCR), as described previously^10^. Briefly, 1 μg of RNA was subjected to DNase (Ambion, Thermo Fisher Scientific) digestion at 37 °C for 30 min followed by DNase inactivation with 5 mM EDTA at 75 °C for 10 min. cDNA was then synthesized from 1 μg DNase-treated RNA in a 20 μl reaction using iScript reverse transcriptase (Bio-Rad, Hercules, CA), according to manufacturer’s protocol. The *Synpo*, *Nphs1*, *Ptgs2* and *Gapdh* mRNAs were quantified by qRT-PCR using the SYBR green method on an iQ5 thermal cycler (Bio-Rad) using forward and reverse primers (Table 2). The PCR conditions were as follows: 1 cycle at 95 °C for 3 min, 40 cycles at 95 °C for 10 sec, and 55 °C for 10 sec, followed by a melt curve analysis. The amplification efficiency of each primer pair was then measured by plotting the efficiency curve of serial dilutions of selected cDNA samples. Values were normalized to the housekeeping gene *Gapdh* and plotted as fold changes relative to vehicle control treatments.

### Western Blotting

Protein extracts (10–20 μg) were separated by SDS-polyacrylamide gel electrophoresis (PAGE), and transferred to nitrocellulose membrane (Bio-Rad). Membranes were blocked for 1 hour with 5% milk in PBS containing 0.1% Tween 20 followed by incubation in primary antibody overnight [anti-phospho-GR (Ser211), anti-PPARγ, anti-phospho Akt (Ser 473) and anti-Akt (Cell Signaling Technology, Danvers, MA); anti-GR (Santa Cruz Biotechnology, Dallas, TX); anti-phospho-PPARγ (Ser 273) (Bioss Inc., Woburn, MA); and anti-β-Actin (Sigma-Aldrich)]. Following four washes, blots were incubated in horseradish peroxidase-conjugated secondary antibodies against mouse or rabbit IgG (Jackson Immuno Research Laboratories, West Grove, PA) for 2 hours at room temperature. Protein bands were detected on X-ray film using the ECL chemiluminescence reagent (GE Healthcare Bio-Sciences, Pittsburgh, PA). X-ray films were scanned using a calibrated ArtixScan M1 transillumination scanner (Microtek Lab Inc., Cerritos, CA) controlled by the ScanWizard Pro program (version 7.042) and densitometric analysis of the integrated band density was performed using ImageJ (version 1.39, standard settings; http://rsb.info.nih.gov/ij/).

### Immunofluorescence Staining

Kidneys were examined by indirect immunofluorescence on 4-micron thick paraffin sections. The sections were deparaffinized with xylene and rehydrated in graded ethanol. Antigen retrieval was performed by boiling the slides in 10 mM sodium citrate followed by PBS-Tween (0.5% Tween-20) wash. Blocking was done with Super Block (Scytek Labs Inc., Logan, UT) for 6 min at 37 °C, followed by incubation with anti-synaptopodin primary antibody (Santa Cruz Biotechnology) in 5% super-block at 4 °C overnight at 1:100 dilution. Sections were washed three times for 10 min each with 2.5% Super Block in PBS-Tween (0.5% Tween-20) and incubated with fluorescent secondary antibody (Alexa Flour, 2 ug/ml; Invitrogen, Carlsbad, CA) in 5% Super Block in PBS for 1 hour at room temperature. Sections were then washed three times for 10 min each with 2.5% Super Block in PBS-Tween (0.5% Tween-20), mounted with Prolong Gold Antifade Reagent (Invitrogen). Kidney sections from the groups were viewed and imaged with exposures within 250–600 ms with an Axio Scope fluorescent microscope (Carl Zeiss Microscopy GmbH, Jena, Germany).

### Statistical Analyses

Statistical differences were determined by the unpaired Student’s *t*-test for single comparisons and two-way ANOVA for multiple comparisons, using the GraphPad Prism software version 6.00 for Windows. *P*-values < 0.05 were considered to be significant and all data are presented as mean ± SEM.

## Additional Information

**How to cite this article**: Agrawal, S. *et al.* Pioglitazone Enhances the Beneficial Effects of Glucocorticoids in Experimental Nephrotic Syndrome. *Sci. Rep.* **6**, 24392; doi: 10.1038/srep24392 (2016).

## Supplementary Material

Supplementary Information

## Figures and Tables

**Table 1:** Proteinuria values for different treatment groups during the study at different days (expressed as UPC ratios).

Group	Proteinuria (UPC; mg/mg) Mean ± SEM		
	Day 0	Day 7	Day 11
Control	2.10 ± 0.34	2.38 ± 0.17	2.01 ± 0.14
PAN	1.38 ± 0.13	8.22 ± 2.63	27.95 ± 8.95*
PAN+low-dose GC	1.34 ± 0.10	8.50 ± 1.89	21.26 ± 6.07*
PAN+high-dose GC	1.34 ± 0.08	4.96 ± 1.06	7.27 ± 2.13^#^
PAN+Pio	1.26 ± 0.09	4.94 ± 0.83	12.06 ± 2.73^#^
PAN+Pio+low-dose GC	1.29 ± 0.12	6.91 ± 2.43	11.52 ± 4.88^#^
PAN+Pio+high-dose GC	1.35 ± 0.18	2.00 ± 0.15	2.72 ± 0.67^#^

^*^P < 0.05 vs. control.

^#^*P* < 0.05 vs. PAN.

**Table 2:** Genes and Their Respective Primers Used in This Study.

Gene	Genbank ID	Forward	Reverse
Synpo	NM_021695	caaacccaacactccaagcg	tgcatgccaatgagcagaga
Nphs1	NM_022628	tgctctttgcagttggtggt	tcctgatcctgtcctccgac
Ptgs2	NM_017232	attgctggccgggttgctgg	tcagggagaagcgtttgcggt
Gapdh	NM_017008	tgtatccgttgtggatctga	cctgcttcaccaccttcttga

Sequences are in 5′ to 3′ direction.
