# Supplementary material for: Pioglitazone Enhances the Beneficial Effects of Glucocorticoids in Experimental Nephrotic Syndrome
Source: Sci Rep. 2016 May 4;6:24392. doi: 10.1038/srep24392 (PMC4855145; doi:10.1038/srep24392)
Supplement: Supplementary Information [file srep24392-s1.pdf]

Supplementary Data

Pioglitazone Enhances the Beneficial Effects of Glucocorticoids in Experimental Nephrotic Syndrome

[*1]S Agrawal, [1]M A Chanley , [2]D Westbrook, [1]X Nie, [1]T Kitao, [1]A J Guess, [1,3]R Benndorf, [2,4]G Hidalgo, and [1,3]W E Smoyer

[1]Center for Clinical and Translational Research,
The Research Institute at Nationwide Children's Hospital, Columbus, OH;
[2]James and Connie Maynard Children's Hospital, Greenville, NC;
[3]Department of Pediatrics, College of Medicine,
The Ohio State University, Columbus, OH;
[4]Department of Pediatrics, Brody School of Medicine,
East Carolina University, Greenville, NC.

Short Title: Repurposing pioglitazone for NS

***Address correspondence to:**
Shipra Agrawal, Ph.D.
Center for Clinical and Translational Research,
The Research Institute at Nationwide Children's Hospital,
Room W323, 700 Children's Drive,
Columbus, OH 43205; Fax: 614-722-3330; Phone: 614-722-6886
E-mail: shipra.agrawal@nationwidechildrens.org

Podocyte Culture and Treatments

Conditionally immortalized murine podocytes (MPC-5) were cultured and differentiated according to a standard protocol, as described previously [1]. The cell line was cultured under proliferating conditions in RPMI 1640 medium supplemented with 10% fetal bovine serum (FBS), 100 U/ml penicillin, 100 μg/ml streptomycin (Invitrogen), and 10 U/ml mouse γ-interferon (Sigma-Aldrich) at 33°C in a humidified atmosphere of 5% CO2. Differentiation was induced in the absence of γ-interferon at 37°C, and the cells were further cultured on coverslips for 10 to 14 days before treatment. Differentiated podocytes were pretreated for 2 h with the vehicle dimethyl sulfoxide (DMSO, Sigma-Aldrich), 0.1 μM pioglitazone (Pio; Enzo Life Sciences International Inc., Plymouth Meeting, PA) and 10 μM glucocorticoid dexamethasone (GC; Sigma-Aldrich) followed by puromycin aminonucleoside (PAN; Sigma-Aldrich) treatment (100μg/ml) for 24 hours. The cells were further assayed for viability (**Figure S1A**) or actin staining (**Figure S1B**) and visualized under a fluorescence microscope.

Viability Assays

Viability assays were performed using the LIVE/DEAD Viability/Cytotoxicity Kit according to the manufacturer's instructions (Molecular Probes, Invitrogen). In brief, cells were washed gently with phosphate-buffered saline (PBS) and incubated with combined LIVE/DEAD assay reagents containing calcein-AM and ethidium homodimer-1 for 45 minutes. The wet coverslips were carefully mounted on the microscope slide with Prolong Gold antifade reagent (Invitrogen), sealed and the labeled cells were viewed under the fluorescence. Images were captured using 10X objective on a Leica DMI6000B inverted fluorescence microscope (Leica Microsystems, Bannockburn, IL) at excitation/emission of 494/517 nm for calcein-AM and 528/617 nm for ethidium homodimer-1. Digital micrographs were captured using a Retiga SRV

14-bit grayscale charge-coupled device camera (QImaging, Surrey, BC, Canada) and the images were processed and merged using Adobe Photoshop CS3 (Adobe Systems, Mountain View, CA).

Actin Staining

Cells were stained with Texas Red–X phalloidin to detect actin stress fibers according to the manufacturer's instructions (Invitrogen). In brief, after treatments, cells were washed twice with PBS, fixed in 3.7% formaldehyde for 10 min, washed twice with PBS, permeabilized with 0.1% Triton X-100 for 5 min, washed 2X with PBS, blocked with 1% bovine serum albumin for 30 min, and stained with Texas Red–X phalloidin for 20 min. Cells were washed twice with PBS, air-dried, and mounted with Prolong Gold antifade reagent containing 4′,6-diamidino-2-phenylindole (DAPI; Invitrogen). Images were captured using a 40X objective on a Leica DMI6000B inverted fluorescence microscope (Leica Microsystems) at excitation/emission of 591/608 nm for Texas Red and 358/461 nm for DAPI. Digital micrographs were captured using a Retiga SRV 14-bit grayscale charge-coupled device camera (QImaging) and the images were processed and merged using Adobe Photoshop CS3 (Adobe Systems).

Pediatric Case Report

Following the addition of pioglitazone to the treatment of a child with refractory NS, the child was monitored for potential drug-induced side effects, proteinuria [measured as urine protein:creatinine ratios (UPC)], serum albumin, echocardiogram evaluations and other routine parameters throughout the study. Prior to the introduction of pioglitazone, the child was monitored for potential drug toxicity due to tacrolimus and Acthar® with serial measurements of HbA1c and fasting lipids. First-AM urine samples were collected and analyzed for urine protein:creatinine (UPC) ratios every two weeks. Serial measurements of serum albumin levels

were also monitored to assess the degree of hypoalbuminemia. Determination of the child's overall immunosuppression was calculated throughout his course based on a semi-quantitative scale developed to track clinically indicated changes in each immunosuppressive medication. A generalizable model to quantitate immunosuppression throughout the treatment was developed (**Table S2A**), which was then applied to this child's specific immunosuppressive medications (**Table S2B**), and the overall immunosuppression calculated at each time point studied and shown as the total Immunosuppression Score (ISS), similar to previous approaches [2,3]. The calculated Total ISS values were based on percentages of the maximum published doses of each immunosuppressive medication prescribed[4].

Pioglitazone Initiation Associated with Marked Reductions in Both Proteinuria and Overall Immunosuppression in a Child with Refractory Nephrotic Syndrome

A four year old boy was referred with steroid resistant NS for a second opinion. Three months prior to referral, his initial treatment was standard therapy with high dose oral prednisone for 6 weeks, although he failed to enter remission. A subsequent renal biopsy revealed minimal change nephrotic syndrome. He was then prescribed cyclosporine but he had poor gastric tolerance, and had to discontinue this medication. On referral, his serum albumin was 2.1 g/dl, serum creatinine was 0.21 mg/dl, total cholesterol was 303 mg/dl, and a random urine protein:creatinine ratio (UPC) was 9.1 (**Figure S2**). Due to the mother's reluctance to administer calcineurin inhibitors, his treatment at referral included only lisinopril. However, due to persistent massive proteinuria, tacrolimus (TAC) was added at week 5 at 0.25 mg PO BID (0.018 mg/kg/day; target trough serum levels of 6-8 ng/dl) and prednisone added at 5 mg PO QAM (5.37 mg/m^2/d). Within 4 weeks his clinical course became complicated by recurrent infections and anasarca, requiring eight hospital admissions and 25 IV albumin infusions over the following

35 weeks. A repeat renal biopsy at week 17 revealed mesangial sclerosis and focal segmental glomerulosclerosis (FSGS). Numerous alternative treatments for NS were initiated over the following year, including high-dose IV steroids, losartan, IV rituximab (RTX) and mycophenolate mofetil (MMF). Later in his course, Acthar® injections were also added to his regimen, but he subsequently developed hypertension, increased hemoglobin A1C, increased appetite and body mass index, more frequent respiratory infections, C. difficile infection, skin hyperpigmentation, tooth enamel discoloration, and hirsutism.

Given his failure to respond to the above treatments and his significant reported side effects, the family was offered experimental treatment with pioglitazone, based on its renoprotective effects and data in this manuscript [5-8]. After receiving exemption from the IRB for the use of pioglitazone and review of potential side effects [9] with the family, informed consent was obtained. Pioglitazone was added to his treatment regimen in week 45 at a dose of 15 mg PO QD, based on a dosage adjustment for his body surface area, available dosage forms, and a recent pharmacokinetics modeling report [10]. As a precaution, baseline and serial echocardiograms, as well as monthly urine cytology screenings, were also performed. After one week on pioglitazone, his UPC had declined from 7.5 to 4.6 mg/mg (38% reduction; **Figure S2**), and no signs of hypoglycemia or other adverse effects were observed. After four weeks on pioglitazone his dose was increased to 30 mg PO QD, and over the ensuing 13 weeks the UPC continued to trend downward to ~3.2 mg/mg (a 57% reduction from pioglitazone initiation; **Figure S2**). The observed decrease in proteinuria was not associated with any decrease in estimated glomerular filtration rate (eGFR; **Figure S3**). Importantly, however, the reduced proteinuria enabled his clinical care team to initiate reductions in his other immunosuppressive medications. Subsequent reductions in his overall immunosuppression were quantitated using a

“Total Immunosuppression Score” (Total ISS) scale we developed to track changes in his overall immunosuppression and its associated risks (**Tables S2A and S2B**). Over the 35 weeks following introduction of pioglitazone, the patient’s UPC gradually decreased even further to 1.49 mg/mg (an 80% reduction from pioglitazone initiation). In addition, these reductions in proteinuria allowed his clinical care team to further reduce or discontinue many of his immunosuppressive medications, which was reflected in a reduction in his Total ISS from 11 to 4 (a 64% reduction from pioglitazone initiation; **Figure S2**). These improvements were also accompanied by a decreased frequency of upper respiratory infections, normalization of his hemoglobin A1C, improvements in weight, body mass index and blood pressure, as well as resolution of his hyperpigmentation, tooth enamel discoloration, hirsutism and acne. In summary, introduction of pioglitazone to the treatment regimen of this child with refractory NS was associated with an 80% reduction in proteinuria despite a simultaneous 64% reduction in overall immunosuppression, as well as reductions in multiple drug-induced side effects, and complete elimination of his previously-required hospitalizations and IV albumin infusions. Importantly, these clinical benefits were realized with no side effects attributable to pioglitazone being observed during his treatment.

Table S1: Total % body weight gain and final average kidney weights of rats in the animal study.

Weight (wt.)	—	+ PAN				
	**Control**	**—**	**low-dose GC**	**high-dose GC**	**Pio+low-dose GC**	**Pio+high-dose GC**
**% body wt. growth ***	164 %	146 %	138 %	132 %	139 %	132 %
**Mean kidney wt. (mg)****	970.9 ± 27.5	1032 ± 24.5	987.3 ± 47.4	953.1 ± 34.1	937.9 ± 37.4	937.5 ± 37.5

* The % body weight growth is the average growth in body weight of all the rats in the indicated control or experimental group at Day 11, starting with a mean weight of 100% at Day 0.

** Mean kidney weight is the average weight of both kidneys from each rat in the indicated control or experimental group at Day 11.

Table S2A. "Total Immunosuppression Score" (Total ISS) Template Design

% Full Dosing	Immunosuppression Score	Drug #1	Drug #2	Drug #X	Total Immunosuppression Score
≥75%	4				
≥50 - <75%	3				
≥25 - <50%	2				
<25%	1				
0%	0				

Table S2B. Personalized "Total Immunosuppression Score" for Presented Case

ISS (% full dose)	Prednisone	TAC	Acthar®	MMF	RTX	Potential Total ISS
**4 (≥75%)**	≥ 46 mg/m^2/d	≥ 0.151 mg/kg/d	≥ 31 units BIW	≥ 901 mg/m^2/d	<6 months after first dose (375 mg/m^2/dose X 1-4 doses)	**20**
**3 (≥50-<75%)**	31-45 mg/m^2/d	0.11-0.15 mg/kg/d	21-30 units BIW	601-900 mg/m^2/d	n/a	
**2 (≥25-<50%)**	16-30 mg/m^2/d	0.051-0.1 mg/kg/d	11-20 units BIW	301-600 mg/m^2/d	6-9 months after first dose (375 mg/m^2/dose X 1-4 doses)	
**1 (<25%)**	≤ 15 mg/m^2/d	≤ 0.05 mg/kg/d	≤ 10 units BIW	≤ 300 mg/m^2/d	n/a	
**0 (0%)**	0 mg/m^2/d	0 mg/kg/d	0 units BIW	0 mg/m^2/d	>9 months after first dose (375 mg/m^2/dose X 1-4 doses)	**0**

ISS= Immunosuppression Score; TAC = Tacrolimus; MMF = Mycophenolate Mofetil; RTX = Rituximab

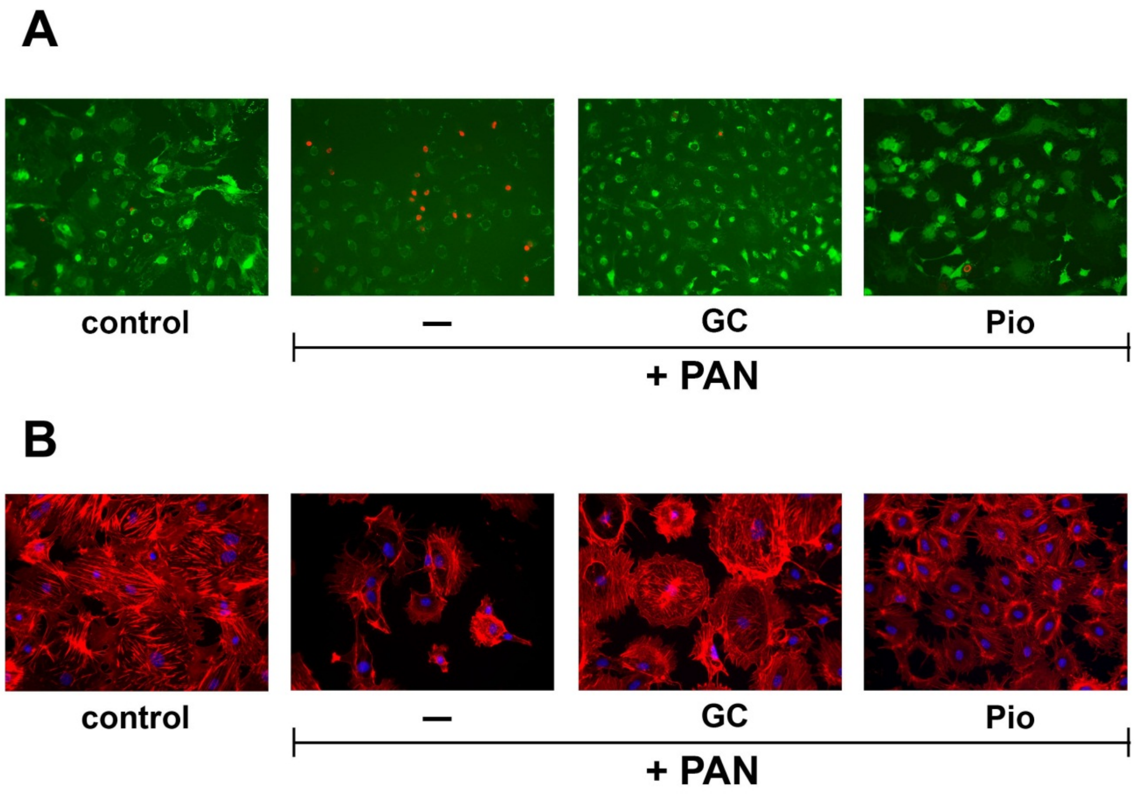

Figure S1. Glucocorticoids and Pioglitazone Protect Podocytes Against Injury.

Treatment with either GCs or pioglitazone enhanced podocyte viability and reduced actin cytoskeletal disruption during PAN-induced injury. Differentiated cultured murine podocytes were pre-treated with vehicle (DMSO), 0.1 µM pioglitazone (Pio), or 10 µM dexamethasone (GC) followed by puromycin aminonucleoside (PAN) treatment at 100µg/ml for 24 hours. The cells were then analyzed for viability/cytotoxicity and actin cytoskeletal rearrangement. **A)** Representative fields showing live cells in green (calcein-AM) and damaged cells in red (ethidium homodimer-1); **B)** Representative fields showing staining of actin filaments in red (Texas Red phalloidin) and nuclei in blue (DAPI).

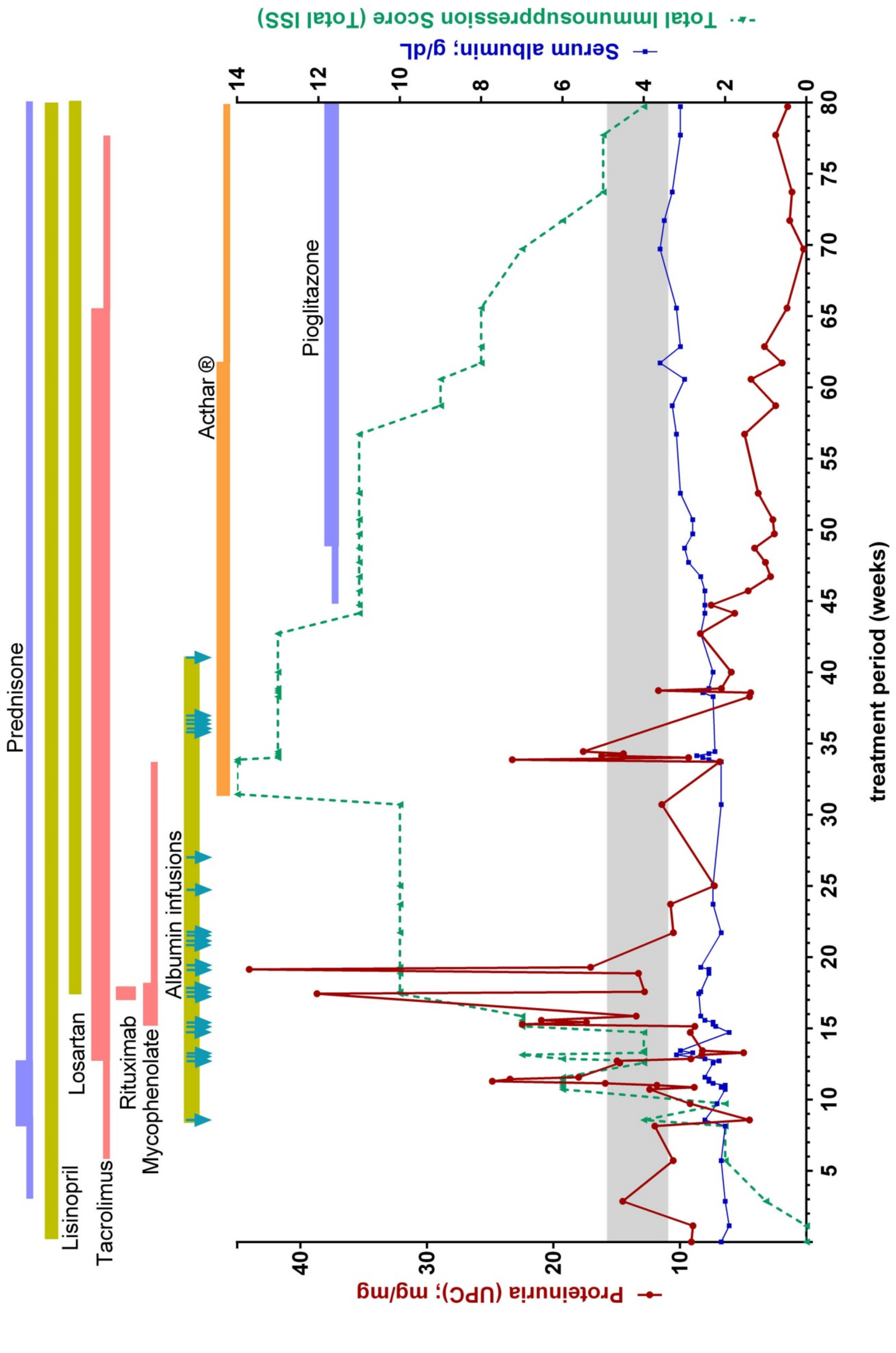

Prednisone
Lisinopril
Losartan
Tacrolimus
Rituximab
Mycophenolate
Albumin infusions
Acthar ®
Pioglitazone
Proteinuria (UPC); mg/mg
Serum albumin; g/dL
Total Immunosuppression Score (Total ISS)
treatment period (weeks)

Figure S2. Pioglitazone Initiation Associated with Marked Reductions in Both Proteinuria and Overall Immunosuppression in a Child with Refractory Nephrotic Syndrome. The graph depicts the treatment regimen and clinical course of a 4 year old child with refractory NS over the course of 80 weeks. During his course he received treatment with multiple immunosuppressive drugs (**horizontal bars at top of figure**), including prednisone, tacrolimus, lisinopril, losartan, mycophenolate mofetil, and rituximab, all without a notable reduction in his proteinuria (**red solid line**). He required eight hospitalizations and 25 IV albumin infusions (**green arrows**) to clinically manage his disease by maintaining acceptable serum albumin levels (blue solid line). The addition of Acthar® to his treatment regimen appeared to moderately reduce his proteinuria, but also increased his Total Immunosuppression Score (ISS) to 14 (**green dotted line**) and was associated with multiple side effects (see Text). The introduction of pioglitazone in week 45 (15 mg PO QD for 4 weeks, followed by 30 mg PO QD for 31 weeks) to his treatment regimen was associated with a gradual reduction in proteinuria by 80%, as well as stabilization of serum albumin levels (3.4-4.9 g/dL; grey shaded area) and elimination of the need for any subsequent hospitalizations or albumin infusions. The proteinuria reduction associated with the introduction of pioglitazone also enabled reductions in the doses of other immunosuppressive drugs, as highlighted by the notable decrease in the Total ISS from 11 to 4 (64% reduction).

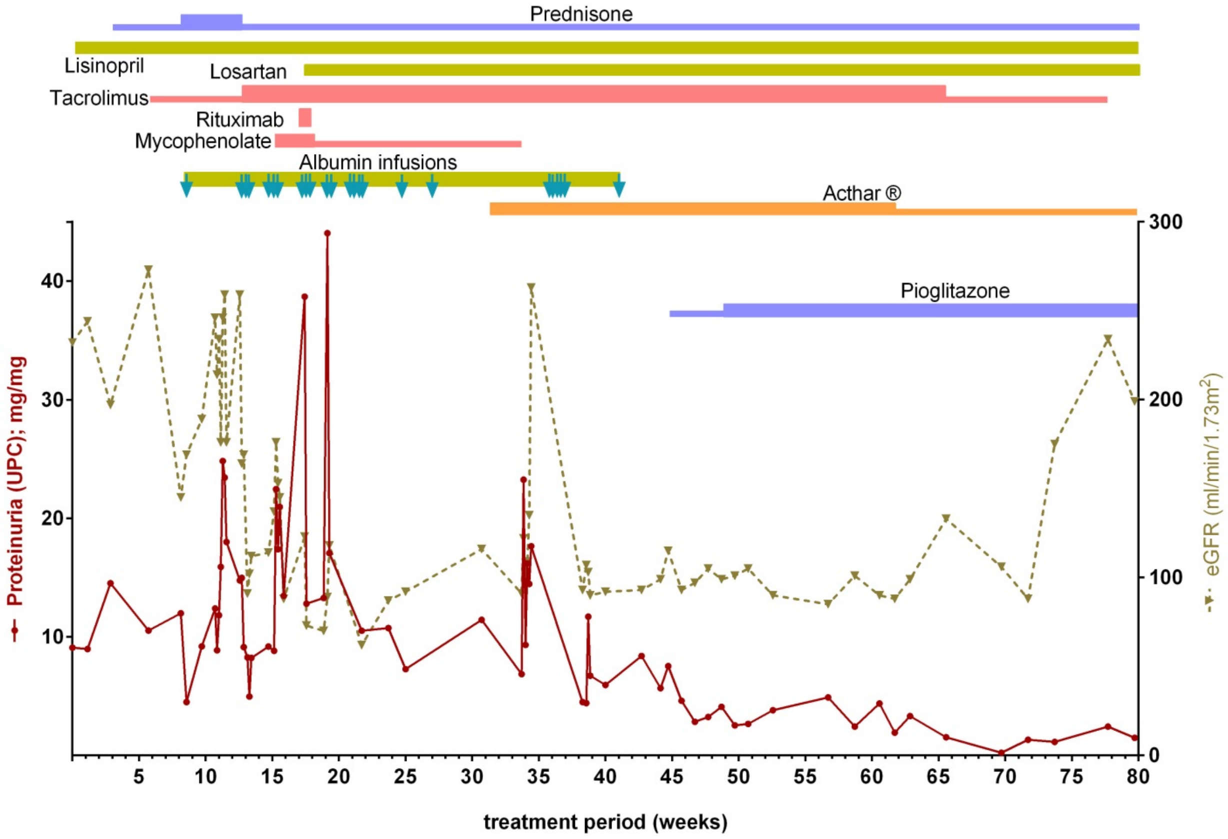

Figure S3. The decrease in proteinuria (UPC) after the addition of pioglitazone was not associated with a decrease in estimated glomerular filtration rate (eGFR).

References:

1. Agrawal, S., Guess, A.J., Benndorf, R. & Smoyer, W.E. Comparison of direct action of thiazolidinediones and glucocorticoids on renal podocytes: protection from injury and molecular effects. *Mol Pharmacol.* **80**, 389-399 (2011).
2. Vasudev, B., *et al.* BK virus nephritis: risk factors, timing, and outcome in renal transplant recipients. *Kidney Int.* **68**, 1834-1839 (2005).
3. Hocker, B., *et al.* Epidemiology and morbidity of Epstein-Barr virus infection in pediatric renal transplant recipients: a multicenter, prospective study. *Clin Infect Dis.* **56**, 84-92 (2013).
4. Gipson, D.S., *et al.* Management of childhood onset nephrotic syndrome. *Pediatrics.* **124**, 747-757 (2009).
5. Yang, H.C., *et al.* The PPARgamma agonist pioglitazone ameliorates aging-related progressive renal injury. *J Am Soc Nephrol.* **20**, 2380-2388 (2009).
6. Yang, H.C., Ma, L.J., Ma, J. & Fogo, A.B. Peroxisome proliferator-activated receptor-gamma agonist is protective in podocyte injury-associated sclerosis. *Kidney Int.* **69**, 1756-1764 (2006).
7. Zuo, Y., *et al.* Protective effects of PPARgamma agonist in acute nephrotic syndrome. *Nephrol Dial Transplant.* **27**, 174-181 (2012).
8. Sarafidis, P.A., Stafylas, P.C., Georgianos, P.I., Saratzis, A.N. & Lasaridis, A.N. Effect of thiazolidinediones on albuminuria and proteinuria in diabetes: a meta-analysis. *Am J Kidney Dis.* **55**, 835-847 (2010).
9. Soccio, R.E., Chen, E.R. & Lazar, M.A. Thiazolidinediones and the promise of insulin sensitization in type 2 diabetes. *Cell Metab.* **20**, 573-591 (2014).

10. Sherwin, C.M., Ding, L., Kaplan, J., Spigarelli, M.G. & Vinks, A.A. Optimal study design for pioglitazone in septic pediatric patients. *J Pharmacokinet Pharmacodyn.* **38**, 433-447 (2011).
